# Correction: Uncoupling the roles of firing rates and spike bursts in shaping the STN-GPe beta band oscillations

**DOI:** 10.1371/journal.pcbi.1013638

**Published:** 2025-11-03

**Authors:** Jyotika Bahuguna, Ajith Sahasranamam, Arvind Kumar

An additional affiliation is missing for the third author. Arvind Kumar is also affiliated with the Science for Life Laboratory, Solna, Sweden.
