# Supplementary material for: Uncoupling the roles of firing rates and spike bursts in shaping the STN-GPe beta band oscillations
Source: PLoS Comput Biol. 2020 Mar 30;16(3):e1007748. doi: 10.1371/journal.pcbi.1007748 (PMC7145269; doi:10.1371/journal.pcbi.1007748)
Supplement: Attachment — Submitted filename: PointByPointReply.pdf [file pcbi.1007748.s013.pdf]

Dear Dr. Marinazzo

We thank the reviewers for their insightful comments and feedback. We have revised the manuscript according to their suggestions. The changes in the manuscript are marked in blue. In the following we provide a point by point reply to the reviewers' questions and provide pointers in the manuscript where corresponding change can be seen. All the new text in the manuscript is also marked in blue.

Best,

Arvind Kumar on the behalf of co-authors

Reviewer #1:

Firing rate vs. firing pattern is a perennial issue in neuroscience. The question of firing rate vs. firing pattern significance in Parkinson's disease has been around for several decades. It was driven by clinical and experimental considerations: spiking units are recorded from patients or parkinsonian animal models, the next step one would do is to see if firing rate, bursting rate (or other measure of phasic and/or oscillatory activity) or both correlates with the symptoms. Over the years, the answer has shifted from firing rate to burstiness to oscillations and synchronization strength. However different studies point to different answers and the situation is somewhat messy. I think the prevailing consensus is that both firing rate and firing patterns are altered in Parkinson's disease, but firing pattern (primarily oscillations and synchrony in the beta-band) are strongly related to some of the parkinsonian motor symptoms.

The issue is that while one may study experimental data for the best correlates of symptoms, firing rate and firing patterns are strongly connected. Uncoupling them in an artificial way in a model, does not clarify the mechanisms of pathological beta oscillations of Parkinson's disease. Real GPe and STN neurons have multiple mechanisms that connect spiking and bursting. For example, both cells express calcium currents and calcium-dependent potassium SK current. Therefore, the firing rate will affect calcium concentration, in turn affecting SK current, which will affect the bursting. The inverse is true as well, the bursting mechanisms will affect the number of spikes. As a result, the modelling framework of this study is poorly suited for investigation of the mechanisms of beta-band oscillations in basal ganglia. This problem (together with some of the modeling and data analysis problems enlisted below) make me think that this study should be rejected by PLOS Computational Biology. Perhaps it may go to some journal interested in computational models, not directly related to experimental neuroscience.

We thank the reviewer for giving a very crisp history of the issues that we have addressed in this manuscript. We agree with the reviewer that the discussion on the topic of firing rate and bursts is quite messy. But we disagree with the reviewer that decoupling firing rate and bursting in a neuron model is not insightful. It is quite the opposite -- the messy situation can only be resolved with suitable computational models. As the reviewer has said, the effects of firing rates and bursting are tightly coupled and it difficult to separate them in experiments. But computational modeling provides a very effective way to isolate the contribution of changes in firing rate and bursts to pathological oscillations. It is correct that in PD both firing rates and spike bursting are altered but this does not mean that we should not separate their individual contributions to pathology. There are different biophysical processes and network interactions that result in changes in spike bursts and firing rate changes. Therefore, it is very important to tease apart the roles of spike rates and spike patterns (bursts). Such an understanding will help in framing appropriate intervention methods to quench the pathological activity. In our manuscript we have demonstrated that indeed spike rates and spike patterns (bursts) changes in the GPe and STN clearly have different effects on the oscillations (Fig2, Fig3, Fig5).

Modeling and data analysis:

Inter-spike interval in a burst in the model is 2ms (first paragraph of the materials and methods). This implies 500Hz firing rate within a burst, which is unrealistically high, especially for GPe. Moreover, there is a firing rate accommodation phenomena within bursts in GPe and STN, which will affect burst duration and frequency.

We thank the reviewer for pointing this out. The intra-burst ISI interval of 2ms was in fact a typo. We set intra-burst ISI to 5ms (which is equal to the refractory period of the neuron). We have now corrected this typo. Additionally, we have also checked if the results for two additional value of intra-burst ISIs (3ms and 7ms - Supp Fig S6 and Fig S7, respectively). The qualitative nature of the results remained unchanged for

both these values of intra-burst ISIs. We agree with the reviewer that the model cannot capture the firing rate accommodation within a burst and now we have included this point with other limitations of the model in the section - “Limitation” [Lines 744-745].

Why GPe and STN both have excitatory inputs and no inhibitory inputs? GPe receives massive inhibitory input from striatal MSN neurons.

These inputs are used to explore the GPe-STN network state space around the baseline firing rates of the GPe and STN neurons. Here the increased inhibition to GPe is represented by a decrease in excitatory drive to GPe. Because the key to the dynamics is the balance of excitation and inhibition, it does not matter how in exact detail is such a balance is achieved. This paradigm is widely used in computational studies to reduce the model complexity e.g. see Rubchinsky et al. 2012 and Ahn et al. 2016.

STN affects GPe not only directly, but also via polysynaptic pathways (thalamocorticostriatal, thalamostriatal). Why these feedback circuits will not affect the results of this investigation?

We agree with the reviewer that STN-GPe circuit would be affected via polysynaptic pathways such as thalamocortical and thalamostriatal. The reason to exclude these connections are: (1) lack of suitable experimental data to constrain the model parameters, (2) increase in circuit complexity and agents influencing the oscillations and (3) such connections introduce long delays and including those raises a host of new questions which are beyond the scope of current investigation. Now that we have understood the role of spikes in this reduced model setting we can (in a future work) investigate the function of poly synaptic interactions. We have mentioned exclusion of such long polysynaptic connections and other biological details in the section “Limitations” [Lines 745-748].

What exactly does entropy Hs do? Does it capture periodicity outside of the beta band? What does it do for sharp and narrow vs broad and large peaks in spectra? Hs is a very important measure of neural activity in this study and its properties should be clarified.

The spectral entropy (Hs) is a way to calculate if the spectral power is uniformly distributed over all frequencies or is confined to certain frequencies. As explained in the methods [Lines 229-243] we assume that the power spectrum is a probability distribution function and therefore we can estimate its entropy using the Shannon’s formula. If the power is confined to a single frequency (band) then spectral entropy will be zero (low) and when power is distributed equally over all the frequencies spectral entropy will be maximum. The method cannot differentiate whether power is distributed as a single broad peak or multiple narrow peaks. The figure below illustrates how spectral entropy is affected in different scenarios (e.g. noisy signals and multiple peaks in the signal). We do not want to include this in the manuscript as such a figure is not necessary but we have provided the git repository link in the paper to the figure and script used to reproduce the figure (Lines 240-243, 270-275)

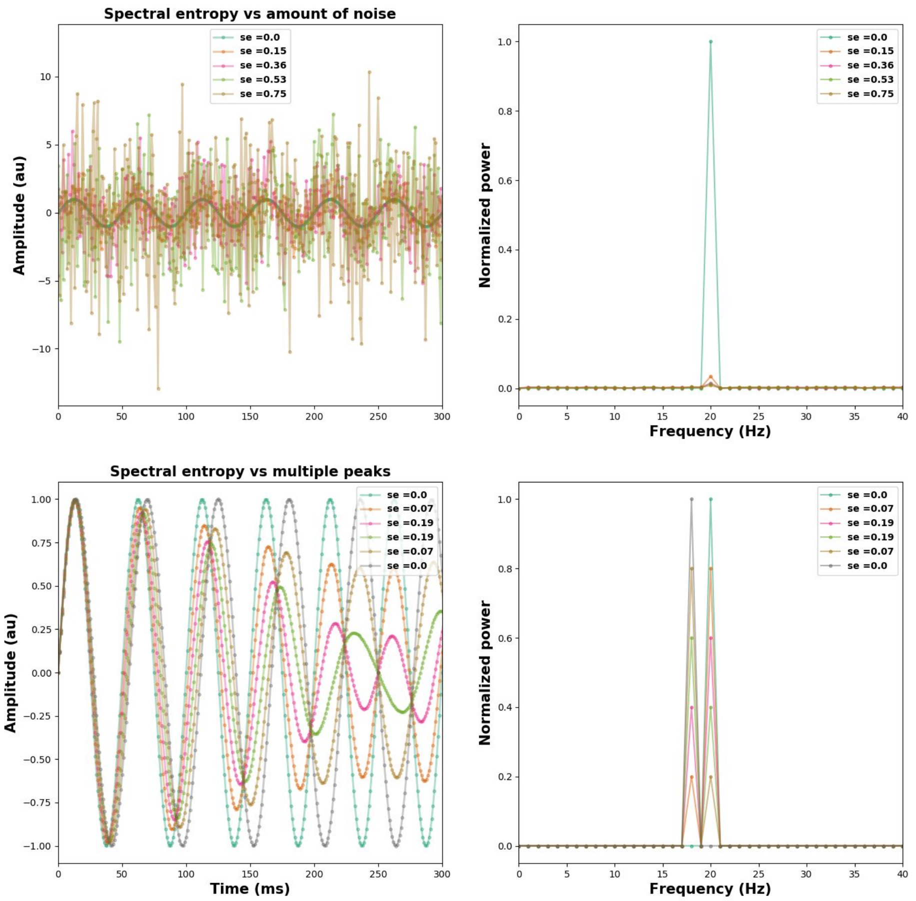

Fig : Example of spectral entropy for an artificial signal. ***top:*** *With increase in noise, the normalized power at the oscillation frequency decreases and hence spectral entropy increases.* ***bottom:*** *The oscillations at 20Hz and 18Hz are mixed with different weights to yield the resultant signal. The "purer" the signal is (single peak - 20Hz), lower is the spectral entropy (se=0.0).*

The effect of non-monotonous dependence of the firing rates etc. – in the inhibitory-excitatory circuit like GPe-STN, these phenomena may strongly depend on the fine-tuning of synaptic coupling. Thus, some robustness study is merited.

We thank the reviewer for raising this question. We have now performed a robustness analysis of the network parameters. While it is not feasible to systematically explore each of the free parameter, we have simulated 10000 different networks for which the parameters were sampled from a normal distribution (mean values as used in Mirzaei et al., 2017; variance = 0.1 x mean value of the parameter) and checked if results shown in Fig 3A can be reproduced qualitatively. The details of the method used are described in the new section - "Robustness analysis of the network parameters" [Lines 187-211] and Lines 302-315 in section "STN firing rate determines the strength of β band oscillations". The distribution of the parameters that meet the criteria (i.e. a good match with results shown in Fig. 3A) are shown in violin plots in Supp Fig S2. Based on these new simulations we can show that our results are robust to parameters variations.

The conclusion of GPe firing rates being not predictive of oscillations is very questionable. The model artificially disconnects firing rates and bursting/oscillations. In real neurons, they are not uncoupled and firing rate change may lead to (or result from) changes in bursting.

We agree with the reviewer that the finding about the role of GPe rates is rather counter-intuitive. But we would like to note that this finding was obtained in a model where we did not have spike bursting neurons (Figure 2), which means it is an effect of purely the change in firing rates. This conclusion is based on the analysis where the resulting STN/GPe firing rates instead of their input drives were used to depict the effect on oscillations (Fig 2E). This is a novel way to visualize the simulation data because it separates whether the change in oscillations were due to decrease in GPe firing rates or a resultant increase in STN firing rates. Such an analysis has not been done before to the best of our knowledge.

But after reading the reviewer's comments, we also checked whether these results hold in the model where STN and GPe neurons are allowed to spike in bursts (Figure 5 in the revised version). In this figure, we compare the effects of firing rate changes due to bursting once the input drive is fixed (note that this isolation is possible due to the neuron model that dis-associates firing rate from bursting) and we observe that for the networks in transition regime, the increase in oscillations is associated with an increase in both STN and GPe firing rates. We discuss this in section "Comparison of firing rate changes due to bursting and input drive" and Figure 5 in the revised version [Lines 457-484].

That the STN firing rates are predictive of oscillations has already been suggested e.g. Tachibana et al. 2011 showed that treating STN with muscimol (blocking STN shows decrease in STN firing rates) and intrapallidal blocking of glutamergic receptors and thereby input from STN (showed a decrease in GPe firing rates) suppressed oscillations whereas intrapallidal blocking of GABAergic receptors (increased GPe firing rates) had no effect on the oscillations (measured as power in the beta band). We have now included this similarity to the experimental results in our text [Lines 343-348]. Furthermore, these results also consistent with the observation that STN firing rates are correlated with motor symptoms of PD (Personal communication: Jose Obeso).

While counter intuitive these observations are very much testable e.g. by simultaneous optogenetic excitation of STN and optogenetic inhibition of GPE (to keep their rate unaffected) can be used to test this prediction. Moreover, we can pool GPe and STN firing rates over different experiments (for a species) and correlate them to the beta oscillation power.

Moreover, contrary to what the manuscript seems to suggest, the experimental observations and modeling of "emergence of beta band oscillations is accompanied by a decrease of firing rate of GPe neurons" do not fit with the manuscript observations of GPe firing rate being not related to oscillations.

We understand that the result seems to be in contradiction to the cited experimental and modeling studies, but to the best of our knowledge no one has studied the effect of GPe firing rates while keeping STN rates constant or vice versa. In the previous experiments and modelling studies it was not ensured that a reduction in GPe rate is not accompanied by a corresponding increase in the STN rates. In fact, if we were to analyse different models of beta oscillations (e.g. Terman et al. 2002, Holgado et al. 2011 and Kumar et al. 2010) we find that in all these models GPe firing rates are not correlated with beta oscillations [Hedelin et al., 2019, IBAGS, Biarritz].

Parkinsonian changes resulting from dopaminergic degeneration may lead to oscillatory activity in different ways. Synaptic projections between GPe and STN are changing. But also cellular properties are changing resulting in firing pattern changes (for example, Luocif Woodhall Sehirli Neuropharmacology 2008). These

dopamine-mediated effects are missing from the model. They can potentially be very important and alter the conclusions of the manuscript.

Indeed, there are a number of biological details that are missing in our model. We have included the lack of those biological details in section “Limitations” [Lines 740-772]. In our model the focus is on the network interactions. Even at the network level, we have focused on a specific subset of interactions -- the interaction between indirect and the hyper-direct pathways. One aspect of dopamine mediated effects are captured in terms of changes in the drive to the GPE (from striatum) and STN (from the cortex). In future work more consequences of other effects of dopamine can also be explored in model. With the modeling framework we have used, one can map the changes in synaptic projections in the state space of changes in firing rates (i.e , what is an equivalent change in firing rates of GPe/STN for a certain change in synaptic weight).

Presentation:

The presentation, especially in the abstract, gives a feeling of a very high confidence of the results as applied to the basal ganglia physiology in vivo. A fair manuscript would rather clarify that the results apply only to the model and whether the model is good or not is not really certain.

We have clearly stated in the abstract that the results are based on “numerical simulations” (in the current version) and “spiking neural network simulations using a novel neuron model” in the older version. We are well aware that this is a computational model and it should be verified in animals models. Indeed, we are confident of our modelling approach and results. Therefore, we also have a high confidence in the conclusions drawn from our results.

Beta band oscillations are indeed correlated with akinesia, but not with tremor. Ref 14 reports correlations of LFP in theta (not beta) band with tremor – first paragraph of introduction.

We thank the reviewer for pointing out this mistake. We have now removed tremor as a symptom related to beta oscillations. (corrected this reference) [Lines 54-55].

While several key modeling studies of oscillations in the basal ganglia are reviewed, other important work in this area is not: Humphries Stewart Gurney J Neurosci 2006, Park Worth Rubchinsky Physical Review E 2011, Merrison-Hort and Borisyuk Frontiers Comput Neurosci 2013.

We thank the reviewer for pointing out these references. We had already cited a more recent work of Rubchinsky et al (2012), but we have now also included Rubchinsky et al 2011 and Merrison-Hort et al 2013. We did not include Humphries et al 2006 in our introduction because in that paper authors studied slow alpha and fast gamma oscillations and not the beta band oscillation. We however, now included this reference in our “Limitations” where we talk about future extensions of the work [Lines 750-752].

Discussion of the beta oscillations origin misses a possibility of multiple mechanisms (Pavlides Hogan Bogacz PLOS Comput Biol 2015 and Ahn Zauber Worth Frontiers Comput Neurosci 2016).

We had already included Pavlides et al 2015, and now also include Ahn et al. 2016 in the model limitation section [Lines 770-771]. We specifically thank the reviewer for the later suggestion which now has been included in the discussion.

Discussion of the birth of oscillations in inhibitory-excitatory networks and ref 32 -- oscillations in inhibitory-excitatory circuit is a half-center oscillator idea, which is probably a century old idea in neuroscience.

It is correct that oscillations in EI networks is an old idea and perhaps we should cite more historical references. Therefore, we have added the reference of the original paper by Wilson and Cowan [Lines 65-68].

There seems to be some confusion about spike bursting vs. oscillations in this manuscript. It would help to clarify how precisely both terms are used here.

We thank the reviewer for pointing this out. Spike bursting refers to the occurrence of a 3-5 spikes in a short succession. It is important to make sure such burst of spikes is not expected assuming a Poisson type spike train with matched firing rate. In our model spike bursting is a single neuron phenomenon. Oscillations, on the other hand, are a population phenomenon in which individual neurons do not have to spike in every cycle of the oscillations. Oscillations are determined by the balance of excitation and inhibition -- primarily determined by firing rate of the excitatory and inhibitory neurons and their synaptic weights.

Here we are interested in understanding how spike bursts (single neuron property) affects network oscillations. So the distinction is clear. However, in the second half of the manuscript we study bursts of beta oscillations. These are short epochs of beta oscillations i.e. when power in beta band is higher than expected. In the literature, such short epoch are referred to as beta bursts. Therefore, we chose to use the same terminology. This does create a confusion. We have now carefully read through the manuscript and clarified it by stating *spike bursts* when we refer to bursts of spikes in a single neuron and *beta-oscillation bursts* when we refer to the short epochs of beta oscillations.

The idea that GPe-STN network may be operating in the regime at the border of oscillatory and non-oscillatory states seems to be very similar (although not identical) to an idea of GPe-STN network operating on the border between synchronized and nonsynchronzied dynamics (Park Worth Rubchinsky Physical Review E 2011). It would make sense to discuss how deep this similarity is.

Yes, we agree with the reviewer. We had already mentioned this similarity in our discussion (lineno: 722, Rubchinsky et al 2012, Park et al 2011). In the revised version we have now added a more detailed comparison with Rubchinsky et al. in the section “Tandem of GPe-STN bursting generates burst of beta-oscillations” (Lines 722-730).

Comparison of the burst duration length in mice and primates on page 10. Healthy mice case is reported, while ref. [13] reports human data in disease. Is it a fair comparison? Also, mice vs. primate data comparison is always a complicated issue.

We agree with the reviewer that it is not fair to compare and mix data from animal models and human patients. And we have tried to not mix the data when possible. Our network connectivity data (Mirzaei et al., 2017) was based on rodent experiments. Therefore, the output firing rate of neurons in the model were matched with the data from rodents (Schmidt et al., 2013, Mallet et al., 2016). We use this to predict the % of GPe and STN neurons that should be bursty to attain the burst length of 0.2 seconds in mice (Bello et al., 2017). Only when it comes to the prediction of spike bursts and beta oscillation bursts that we mix the data to some extent. But positive correlation between beta oscillation burst length and amplitude has been observed not only in humans but also in non-human primates and recently in 6-OHDA lesioned rats (Cagnan et al., 2019). The presence of such a relationship between beta oscillation burst length and

amplitude in healthy rats would hence be a prediction of this study. We have now clearly specified this and correlated the reference [Line 514-518].

Discussion of how the manuscript result can explain experiments with DBS does not appear to be convincing given how complex DBS is with stimulation engaging multiple fibers etc. (also, citation for STN firing rates there is missing).

We agree with the reviewer however, we refer to DBS only in the discussion to suggest how DBS might change the effective excitation/inhibition of the circuit [Lines 609-612].

Conclusion of the “Effects of changes in spike bursting…” section in the discussion: “we propose that increase in STN bursting might play a compensatory role…” The increase in bursting may be due to the lack of dopamine (see a ref to Luocif paper above and other experiments). Which appear to be very basic dopamine-mediated phenomena on a cellular level, not related to compensation of network changes.

We agree with the reviewer, the lack of dopamine might underlie the increased bursting in the STN. We found that when spike bursting is increased in both GPe and STN, spike bursts in STN tend to reduce oscillations. Therefore, it justified to suggest that moderate amount of spike bursts in the STN may serve a compensatory role and reduces beta band oscillations. So even if the change in STN bursting is due to a cellular phenomenon, it has a compensatory effect on the oscillations. We have tried to make it clearer in the sentence [Lines 676-677]. Irrespective of whether bursting is a cellular phenomenon or not, we comment only on the effect of such bursting on the network dynamics.

Referencing to supplementary figures in the manuscript is broken.

We thank the reviewer for pointing this out. It has now been corrected.

Reviewer #2: The manuscript "Uncoupling the roles of firing rates and spike bursts in shaping the GPe-STN beta band oscillations" by Bahuguna and colleagues presents a comprehensive computational study trying to untangle the effects of two typical PD phenomena, the change in firing rate and bursting, on beta band oscillations. The study utilizes the State-dependent Stochastic Bursting Neuron (SSBN) which the group used in previous studies to separate the two components.

Major comments:

1. The effect is only in a small regime (osc/non-osc border). It is unclear whether this regime is relevant at all to the physiological state during PD.

We agree with the reviewer that one might get the impression that this effect may not be relevant in a physiological state given that we see the effect of spike bursts only in the border regions. First of all we would also like to note that only after studying the model, we can find out whether the effect is visible in a narrow of broad regime. Second, we would like to argue that the model is indeed operating in a physiologically relevant state. For instance, the firing rates in this regime corresponds to the firing rates recorded during healthy conditions (section "Effect of bursting in STN neurons on beta-band oscillations" line no: 446-448). Furthermore, we show that in this regime we can replicate the burst length recorded in healthy mice in this regime (section: "Control of amplitude and duration of beta-band oscillation bursts by spike bursting"). Both these suggest that the model if operating in a regime consistent with the physiological state during PD. Therefore, even if it is a narrow region it seems to be physiologically relevant. Finally we show that the qualitative results are robust across parameters when sampled within 10% of their values (new section - "Robustness analysis of the network parameters" [Lines 187-211] and Lines 302-315 in section "STN firing rate determines the strength of β band oscillations").

2. The definition of beta is not consistent in the manuscript and varies across analyses, all the way from 10-35 to 15-30 and finally 15-20 (for example in beta bursts) while in the few figures that show the actual oscillations it seems like a narrow band around 20 Hz (or 15 Hz). Consistency and figures with less-process spectrograms are required.

We thank the reviewer for pointing this out. We start with a wider band because we span across a wide range of input firing rates and wanted to account for both low and high beta band. In order to measure the beta bursts however, we restrict to a narrower range because the peak frequency lies around 15-20Hz. We have now clearly mentioned this in the methods [Lines 234-235, 253-256].

3. The analysis of the spectrum utilizes the binned population, why weren't individual neurons examined?

We thank reviewer for this question. We have now included the spectrograms of the activity of individual neurons (Supp. Fig S12) with varying levels of firing activity. We have chosen three neurons with firing rate lower than, equal to and higher than the population mean. These spectrograms show that results are consistent with the earlier results. Spectrogram of neuronal activity, also give some new insights e.g. a neuron does not necessarily participate in every cycle of the oscillation. This suggests that the network exhibiting stochastic oscillations at population level while spiking activity is in SI (Synchronous Irregular) regime. We included the description of the new figure in section "Control of the amplitude and duration of β band oscillation bursts by spike bursting", [Line: 534-540].

4. Results, Figure 3: It seems that the change in bursting changes the rate as well (which might be the actual one changing the oscillations). Isn't this point the exact one that was supposed to be avoided?

We thank the reviewer for pointing this out. Indeed there is a change in firing rate due to bursting, but because of this neuron model, we can now isolate the effect of change in firing rate (and hence on

oscillations) due to bursting as opposed to changes in firing rates due to input drive. However, a figure to compare these changes was missing and therefore we have now added a new section ("Comparison of firing rate changes due to bursting and input drive") and Figure 5 in the revised version. Moreover, with this method, we can map the changes in firing rates due to bursting back to the spectral entropy analysis we did purely based on firing rates (Figure 5 in the revised version). It can be observed that the firing rate changes due to bursting are much smaller than those by the input drives. But more importantly, it led to the insight that it is the firing rate changes due to external input that defines the state of the network, whereas the firing rate changes due to bursting performs the fine tuning over it.

5. It is unclear to what extent the bursting of the model SSBN resembles those of actual GPe and STN neurons.

The model of spike bursts is indeed simple for instance we have assumed that the number of spikes in a burst, inter-spike-interval of spikes within the bursts is constant. Also, there is no biophysical mechanism in our model to generate the spike bursts. But still, the spike bursts parameters are matched with the data i.e. within burst inter-spike interval = 5ms. And we ensured that even in the presence of spike bursts, average firing rate of the neurons were matched to healthy or PD states. To check the sensitivity of our results to the spike burst parameters, in the revised version of the manuscript, we have also investigated the effect of slow and fast bursts (i.e. when inter-spike-interval of spikes within the bursts was 3ms, 5ms or 7ms - Supp Fig S6 and Fig S7). However, this simplistic bursting characteristics is indeed a limitation of the model (as described in section "Limitations") and should be extended in a future work to include more realistic bursting characteristics.

6. Beta oscillations differ greatly between rodents and primates. However, in the current study the computational results are correlated with mixed species experimental results.

This network was tuned for rats (Mirzaei et al., 2017) such that the neurons had a firing rate distribution measured in the rat data (Schmidt et al. 2013, Mallet et al., 2016). We use this to predict the % of GPe and STN neurons that should be bursty to attain the burst length of 0.2 seconds in mice (Bello et al., 2017). Thus, for most of the study we have focused on rodent data. When it comes to predicting the spike bursts during beta oscillations bursts; and correlation among beta oscillation burst parameters, that we try to relate our results to human and non-human primate data. The positive correlation between burst length and burst amplitude has only been observed in human and non-human primates and recently in 6-OHDA lesioned rats (Cagnan et al., 2019). The presence of such a relationship in healthy rats is an assumption of this study. We have now clearly specified this [Lines 514-518].

Minor comments:

1. Introduction, first paragraph: Death of the DA SNc neuron is only part of the story in PD (see Braak & Braak). The cognitive deficits and some behavioral symptoms are related to other processes associated with the disorder.

We thank the reviewer for pointing this out. We now extended the description of the causes of PD and have included the suggested the reference [Lines 42-45].

2. Introduction, first paragraph: Tremor is typically associated with non-beta oscillations.

We thank the reviewer for pointing this out. We agree and apologize for this error. We have now corrected this [Lines 54-55].

3. Introduction, third paragraph: The definition of bursting and its separation from oscillations is not well defined in many of the rodent and primate studies mentioned.

We thank the reviewer for pointing this out. The distinction was indeed unclear. In order to address this we have included a paragraph in the “Introduction” (Lines: 81-90).

4. The observed shifts in oscillation from 20 to 15 Hz are not observed experimentally. Moreover, perhaps the wide beta range in the study comprises of multiple smeared narrow bands?

We thank the reviewer for this question. It encouraged us to look deeper into the literature to address this issue. There is an evidence of drift from higher beta frequencies to lower beta frequencies during a beta-burst, but measured in striatum LFP when treated with a cholinergic agonist (McCarthy et al 2011). But maybe, it is more an issue of high and low beta bands. It has been suggested that the low beta oscillations are anti-kinetic and gets regulated by dopamine whereas high beta oscillations maybe non-pathological in nature (Marceglia et al., 2006, Hammond et al., 2007, West et al., 2018). Our model suggests that the proportion of bursty GPe and STN neurons might affect the oscillation frequency and hence this aspect needs to be explored further. We have included in the section “Limitations” [Lines 750-761].

Reviewer #3: Bahuguna and colleagues aim to dissociate the impact of firing rate and spiking patterns such as bursting on STN-GPE oscillations in the beta frequency band. These insights are quite important since beta oscillations play a central role in both physiological processes (e.g. motor control and impulsivity) and common neurological disorders (e.g. Parkinson's disease).

Extensive work has been conducted on the oscillatory dynamics of the basal ganglia with a particular emphasis on the STN-GPe loop. However, this is one of the first works aiming to disentangle firing rate and bursting firing patterns. Authors also discuss implications of their work in the context of high conflict tasks (i.e. hold your horses) extending the scope of the paper beyond movement disorders.

The methodology is sufficiently explained, and the manuscript is well organized and clearly written. While I thoroughly enjoyed reading this paper, there are couple of points which would significantly improve the paper:

We thank the reviewer for the encouraging comments.

1) Inputs to the STN-GPe populations are uncorrelated Poisson processes and external inputs via the hyperdirect pathway and the indirect pathway are assumed to be tonic excitation and inhibition, respectively. In the context of Parkinson's disease both neural populations are driven by rhythmic inputs and spiking activity are observed at certain phases of these oscillatory inputs (i.e. cortical beta). This issue is not only relevant for Parkinson's disease but also for high conflict tasks. Several experimental studies have shown that during these tasks the mPFC exhibit rhythmic activity patterns (evoked in the theta band and ongoing oscillations in the beta band) and the STN spiking occurs at certain phases of these oscillations. While the ideal scenario would be for the authors to incorporate rhythmic inputs into their model, since this would be a significant undertaking and completely alter the paper - at the very least, the authors should discuss their work in the context of this literature and motivate the simplifications employed in their study.

We thank the reviewer for pointing this out. We also appreciate the reviewer's decision to acknowledge that exploring the non-poissonian input will be a significant undertaking. In the section "Limitation", now we have added new text to discuss the how rhythmic inputs may interact in the STN-GPe network [Lines 768-772].

2) Percentage of neurons that exhibit bursting spiking patterns is an important parameter in this study. The authors should discuss how neurons are recruited to exhibit these firing patterns (not how this switch is implemented but how it would take place in the context of the wider network).

We thank the reviewer for this question we now discuss the recruitment of these neuron in section "Control of amplitude and duration of beta-band oscillation bursts by spike bursting" (Lines: 543-558).

Minor comment:

1) Reference missing – second paragraph of the discussion

Thank you very much for pointing this out, we have now corrected and included the reference.
