# Supplementary material for: Uncoupling the roles of firing rates and spike bursts in shaping the STN-GPe beta band oscillations
Source: PLoS Comput Biol. 2020 Mar 30;16(3):e1007748. doi: 10.1371/journal.pcbi.1007748 (PMC7145269; doi:10.1371/journal.pcbi.1007748)
Supplement: Attachment — Submitted filename: Point_by_point_reply_round2.pdf [file pcbi.1007748.s014.pdf]

Reviewer #1: The new version has been substantially revised. Many concerns have been extensively addressed. However, my major concern is that the study is trying to dissociate the phenomena that are inherently connected at the experimental level. In a modeling study one can isolate firing rate and firing pattern and explore their effects. I don't see how relevant this would be to experimental context, where the firing rate and pattern are tightly connected on a very fundamental level. This is why, as I wrote earlier, this modeling approach may be an interesting exercise in computational modeling, but is remote from experiment. Therefore I don't really see it as a PLOS CB paper. This is not a wrong study as a modeling exercise. But not more than that. I would expect to see it in a modeling journal or maybe PLOS One, which does not emphasize significance and experimental relevance.

We would like to reiterate our point that computational modeling can help understanding neuronal and network mechanisms better by achieving exactly what is not achievable in experiments. In this study, by isolating their individual effects on the oscillations using suitable computational models we provide a basis for altering/controlling beta-band oscillations by experimental manipulation of firing rate or spike bursting alone.

Reviewer #2: The authors addressed most of the points raised regarding the original manuscript in a satisfactory manner. Two issues still need to be expanded:

1. The manuscript refers to multiple sources of physiological data mixing recording conditions such as in-vivo and in-vitro studies and species such as mice, rats, non-human primates and primates. There is a huge difference (much larger than the 10% variability tested for the model) between rodent species such as mice vs. rats and between different animal models of PD. It is crucial to separate the results and their implementation in the model and to state the data sources and differences clearly (results section) and discuss their implications on the model (discussion section).
2. The same issue still exists with beta band definition which is different in different species and (animal) models of PD. The authors should clearly state the relation to the specific beta band that they choose and their reasoning for it.

We thank the reviewer for pointing this out. In this case most of the parameters corresponding to the model were sourced from rodents. We have now clarified this and specified the animal model and experimental conditions corresponding to the observations in various places in the manuscript [marked in blue color].

We have also added a paragraph citing sources for various beta peak frequencies and ranges as measured in rodents. [see lines 236-244].

Next, even though the model was constrained (when possible) by data from rodents, some of the model results are consistent with experimental data measured form non-human primates and human patients. We think that this reflects both the generality of our model and generality of the experimental phenomena. The reviewer is however right to point out that we were indeed extrapolating our results to observations in humans and non-human primates without specifying

the source. Again to clarify we have now corrected this and we have clearly stated the animal model and experimental conditions which match with our model results. In addition, according to your suggestion we have also added a short paragraph to clarify this [see lines 787-793].

Finally, to address the concern that 10% std. of the parameter values may not cover the parameter variance across different animal models, we have increased the std. of the parameters to 20% of their mean value. These new results are consistent with our previous results. We additionally added a subfigure in supplementary Fig S2 showing the peak frequencies generated in these models [see modified supplementary figure S2 - the figure is reproduced in this document below]. With this wide range of parameters, the peak frequency of beta oscillations is centered around ~ 20Hz. We however could not find a study that systematically addresses variability in parameters for rodent species and different animal models of PD.

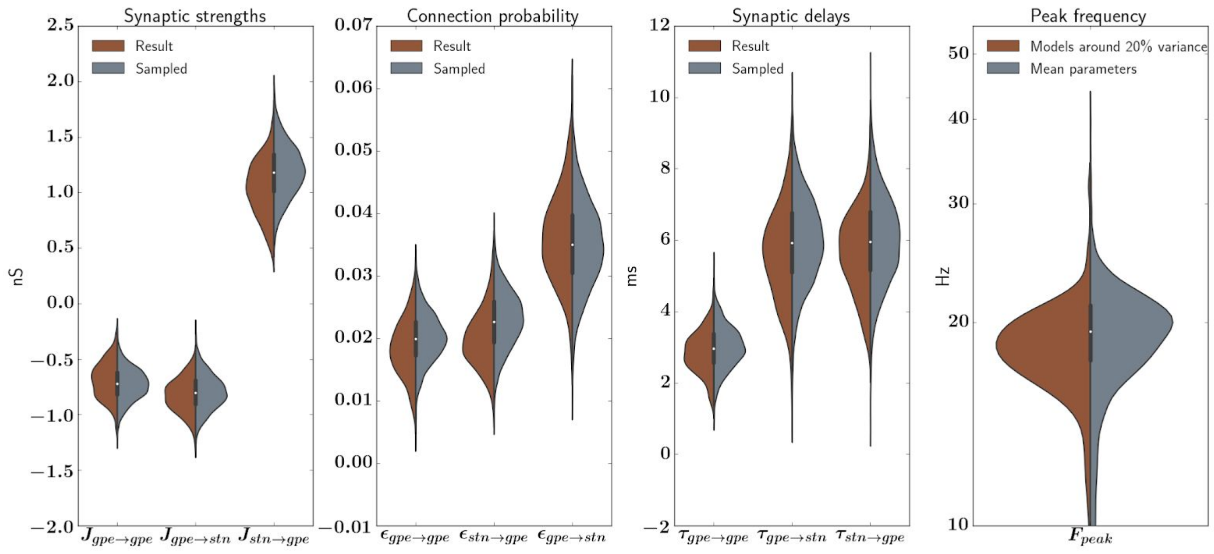

Reviewer #3: Authors have addressed all my concerns .

Thank you for your comment.
